# Expression of CCAAT/Enhancer Binding Protein Beta in Muscle Satellite Cells Inhibits Myogenesis in Cancer Cachexia

**DOI:** 10.1371/journal.pone.0145583

**Published:** 2015-12-28

**Authors:** François Marchildon, Émilie Lamarche, Neena Lala-Tabbert, Catherine St-Louis, Nadine Wiper-Bergeron

**Affiliations:** 1 Graduate Program in Cellular and Molecular Medicine, Department of Cellular and Molecular Medicine, Faculty of Medicine, University of Ottawa, Ottawa, Ontario, Canada; 2 Department of Cellular and Molecular Medicine, Faculty of Medicine, University of Ottawa, Ottawa, Ontario, Canada; University of Minnesota Medical School, UNITED STATES

## Abstract

Cancer cachexia is a paraneoplastic syndrome that causes profound weight loss and muscle mass atrophy and is estimated to be the cause of up to 30% of cancer deaths. Though the exact cause is unknown, patients with cancer cachexia have increased muscle protein catabolism. In healthy muscle, injury activates skeletal muscle stem cells, called satellite cells, to differentiate and promote regeneration. Here, we provide evidence that this mechanism is inhibited in cancer cachexia due to persistent expression of CCAAT/Enhancer Binding Protein beta (C/EBPβ) in muscle myoblasts. C/EBPβ is a bzip transcription factor that is expressed in muscle satellite cells and is normally downregulated upon differentiation. However, in myoblasts exposed to a cachectic milieu, C/EBPβ expression remains elevated, despite activation to differentiate, resulting in the inhibition of myogenin expression and myogenesis. *In vivo*, cancer cachexia results in increased number of Pax7+ cells that also express C/EBPβ and the inhibition of normal repair mechanisms. Loss of C/EBPβ expression in primary myoblasts rescues differentiation under cachectic conditions without restoring myotube size, indicating that C/EBPβ is an important inhibitor of myogenesis in cancer cachexia.

## Introduction

Cachexia causes profound weight loss and muscle mass atrophy, and occurs concomitantly with various diseases including sepsis, AIDS, drug addiction, and cancer [1, 2]. Although often coupled with anorexia, the weight loss and muscle protein catabolism seen in cachectic patients cannot be reversed by nutritional supplementation and thus cannot be attributed to poor nutritional intake alone [3]. Up to 80% of all cancer patients will have cachexia in the advanced stages of their disease [4–6], and this correlates with frailty, increased morbidity and poor outcomes. Further, approximately 30% of cancer deaths are attributed to cachexia rather than tumor burden, making cancer cachexia an important cause of mortality in North America [1]. As such, the creation of treatment and prevention strategies is of utmost importance as cachexia is inversely correlated with the success of anti-cancer treatments and with patient survival [3, 7–9]. The cause of cancer cachexia is thought to stem from a combination of digestive (dysphagia and dysgeusia), humoral (systemic inflammation), endocrine and tumor-derived factors [10–13]. Both cachectic cancer patients and animal models of cachexia have elevated proinflammatory cytokine (e.g. TNFα, IL-1, IL-6) expression in the bloodstream, suggesting that these factors play a role in the development of cachexia [14–18].

In a healthy individual, muscle mass homeostasis is achieved by balancing muscle protein catabolism with protein synthesis. Skeletal muscle atrophy, as seen in cancer cachexia, is mediated at least in part by the upregulation of ATP-dependent ubiquitin E3 ligases (MuRF, MAFbx/atrogin-1) which stimulate the degradation of muscle structural proteins by the 26S proteasome and directly inhibit the translational machinery [19, 20]. Pro-cachectic cytokines stimulate the expression of these ligases and thereby promote increased muscle protein turnover [18, 21–23].

Satellite cells are the primary source of regenerative capacity for skeletal muscle and are found between the sarcolemma and the basement membrane of differentiated muscle fibers [24]. These cells can be activated to both proliferate and differentiate in response to external stimuli, most importantly muscle injury and exercise [25]. Satellite cells are defined by their expression of CD34^+^ and Pax7, among others [26, 27]. As satellite cells differentiate, they progressively lose expression of Pax7 and express in a coordinated fashion the myogenic bHLH factors MyoD, myogenin and MRF-4 that are responsible for the induction of myocyte-specific genes [28]. In muscle wasting conditions, there is a reduction of MyoD expression such that in addition to hypercatabolism, decreased regeneration has been implicated in the pathogenesis of cachexia, linking the inhibition of MyoD expression and reduced differentiation capacity to the development of cachexia *in vivo* [29–31].

CCAAT/Enhancer Binding Protein beta (C/EBPβ) is a bzip transcription factor involved in many regulatory and differentiation processes as both an activator and a repressor. For example, it is required for liver regeneration, acts as a potent commitment factor for adipocyte differentiation, and regulates the acute phase response of the immune system [32–36]. In addition, we have shown that C/EBPβ is also a major regulator of mesenchymal stem cell fate in tissue culture models where it acts as an activator of adipogenesis and a repressor of osteoblastogenesis [37–39]. In healthy muscle, C/EBPβ expression is restricted to Pax7^+^ satellite cells and its expression decreases upon activation [40–42]. Ectopic C/EBPβ expression inhibits myogenesis through inhibition of MyoD protein expression, leading to reduced myogenin and MyHC expression and decreased fusogenic activity [41]. *In vivo*, sepsis, cancer and glucocorticoids can upregulate the expression of C/EBPβ in muscle, as well as trigger cachexia [43, 44]. Indeed, cachexia can stimulate C/EBPβ expression in muscle fibers leading to the expression of atrogin-1 and fiber atrophy [45]. Grafting of the Lewis Lung Carcinoma (LLC) tumor into C/EBPβ null animals prevented the stimulation of atrogin-1 in muscle fibers and muscle atrophy [45], suggesting that loss of C/EBPβ can inhibit muscle atrophy in cancer cachexia. However, the nullizygous C/EBPβ animal is immunocompromised, and thus is unlikely to have normal responses to tumor grafting including cytokine production required for the development of cachexia [46, 47], suggesting that conditional models would provide greater depth of understanding. Further, the nullizygous model cannot distinguish the role of C/EBPβ in the muscle fiber versus potential effects in the satellite cell population driving regeneration.

Multiple lines of evidence indicate that the inhibition of skeletal muscle regeneration, whether through the loss of myogenic precursors, the inhibition of MyoD or through activin type-2 receptor activation, participates in the development of cancer cachexia [31, 48–50]. Herein, we show, using models of cancer cachexia, that stimulation of C/EBPβ expression in satellite cells and myoblasts by the cachectic environment prevents the myogenic regenerative response.

## Results

### Conditioned medium from human cancers induces C/EBPβ expression in myoblasts

**Fig 1:**
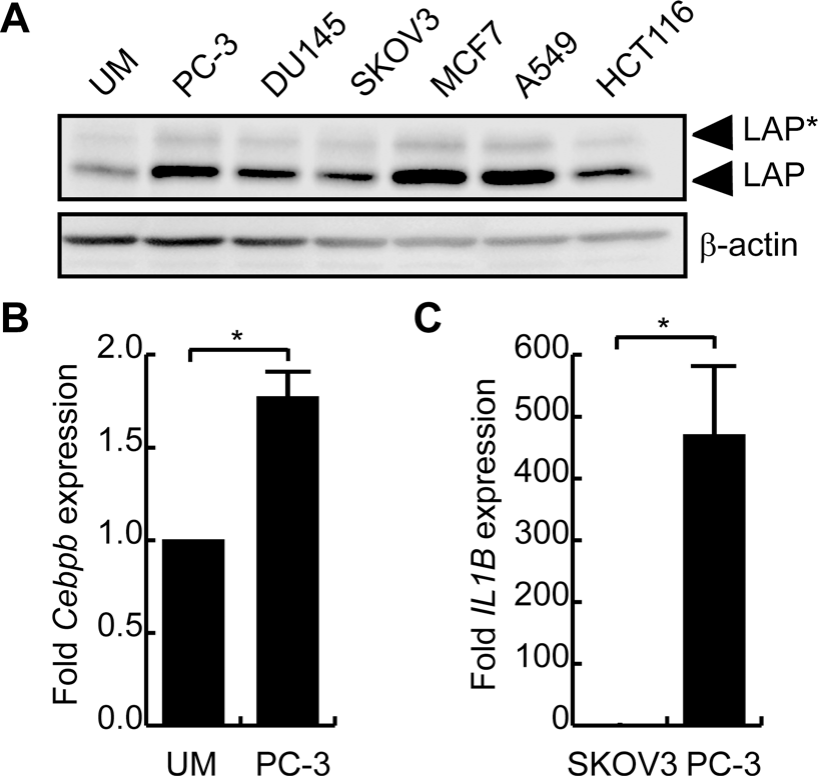
Conditioned medium from human cancers stimulate C/EBPβ expression in myoblasts. **(A)** C2C12 myoblasts were incubated with conditioned medium from indicated human cancers or unconditioned medium (UM) mixed 1:1 with fresh myoblast medium for 48 hours. C/EBPβ expression was assessed by western blot. β-actin is a loading control. **(B)** *Cebpb* mRNA expression in myoblasts treated with PC-3 medium or unconditioned medium for 48 hours. *p<0.05, n = 5. **(C)** *Il1b* expression in SKOV3 and PC-3 cancer cells. *p<0.05, n = 5.

Given that C/EBPβ is an important regulator of the immune system and is downstream of numerous cytokine signaling pathways, we predicted that conditioned medium from human cancers would stimulate C/EBPβ expression in myoblasts. C2C12 myoblasts were cultured with conditioned media (CM) from various human cancer cell lines for 2 days and C/EBPβ expression was assessed by western blotting. Incubation of C2C12 cells with CM from all the tumors tested stimulated C/EBPβ expression to varying degrees, with PC-3, MCF7 and A549 lines producing the most robust increase in expression (Fig 1A). By contrast, the ovarian cancer cell line SKOV3 and the human colon cancer line HCT116 stimulated C/EBPβ expression the least. Incubation with PC-3 CM also stimulated *Cebpb* mRNA expression by almost 2-fold (Fig 1B). Interestingly, PC-3 cells express TNFα, IL-1β and PIF mRNA, and C/EBPβ expression has been shown to be positively regulated by IL-6, a cytokine whose expression is upregulated by IL-1 and TNFα signaling [15, 51–53]. Indeed, while PC-3 cells express *Il1b*, the transcript was undetectable in SKOV3 cells, suggesting a mechanism to explain the differential stimulation of C/EBPβ expression in myoblasts by these two cancer cell lines (Fig 1C).

### Tumor conditioned medium inhibits myogenesis

**Fig 2:**
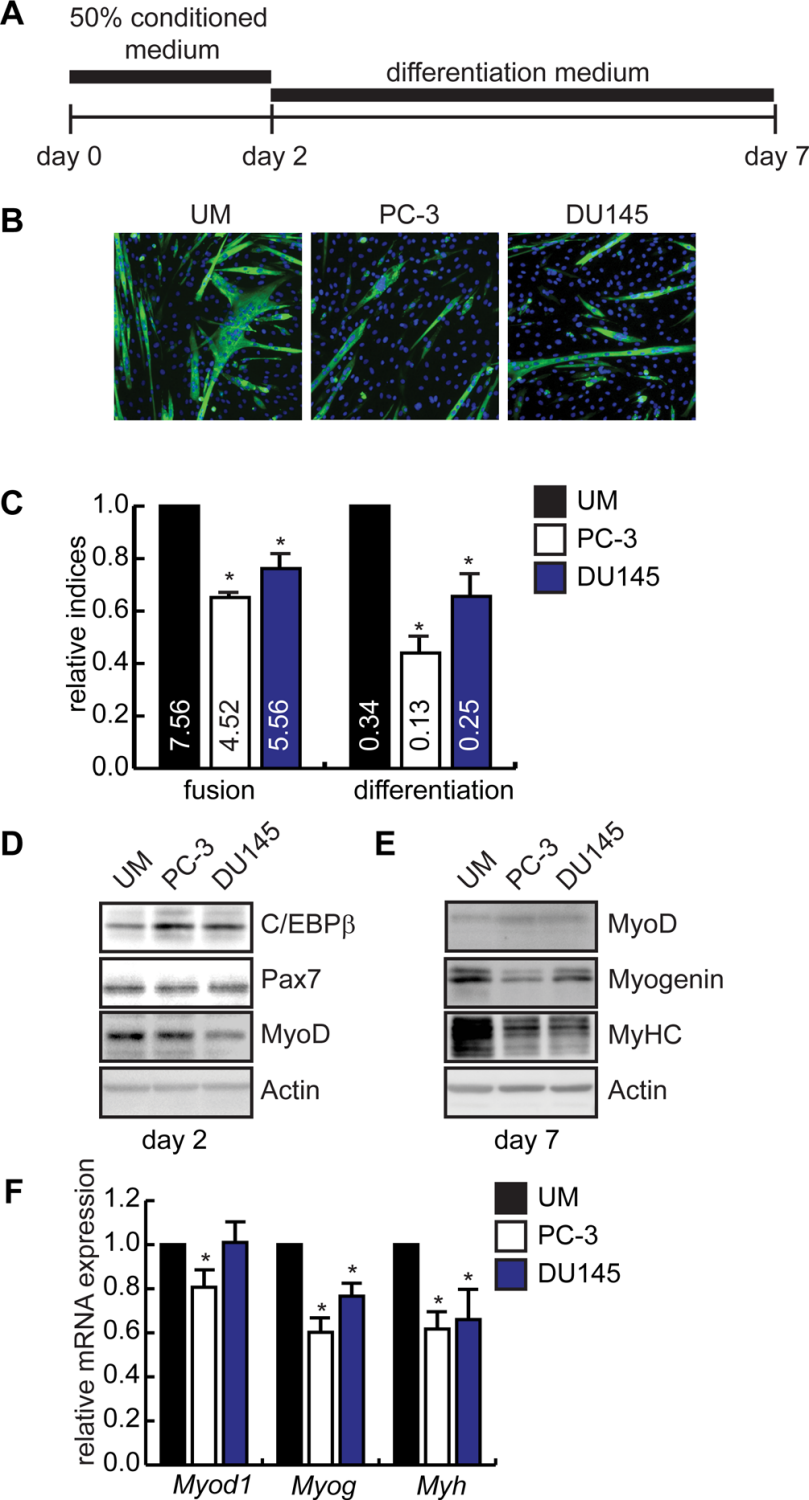
Pre-treatment with conditioned media from a prostate tumor inhibits skeletal muscle differentiation and upregulates C/EBPβ expression. **(A)** Schematic of the treatment procedure for the tissue culture model of cachexia. Conditioned media from PC-3 cells was mixed 1:1 with fresh media, and was added onto proliferating C2C12 myoblasts for 48 hours after which cells were induced to differentiate in fresh DMEM containing 2% horse serum for 5 days. **(B)** Immunocytochemistry staining for myosin heavy chain expression in C2C12 cultures treated with conditioned media from PC-3 or DU145 prostate cancers or unconditioned media (UM) as in (A). DAPI stains nuclei blue. **(C)** C2C12 cultures were induced to differentiate as in (A) and the fusion index (#myonuclei/myotube) and differentiation index (#myonuclei/# total nuclei) was calculated, and shown relative to UM. Actual values are shown in the bars. *p<0.05, n = 7. **(D)** Western blot analysis of C/EBPβ, Pax7, and MyoD expression in proliferating C2C12 cells on day 2 after incubation with conditioned medium. Actin is a loading control. **(E)** Western blot analysis of myogenic marker expression in differentiated C2C12 cells on day 7. Actin is a loading control. **(F)** qRT-PCR analysis of *Myod1*, *Myog* and neonatal myosin heavy chain (*Myh1*, *Myh2*, *Myh8*, *Myh13*) expression, shown relative to cells treated with unconditioned medium (UM) on day 7. *p<0.05, n = 4.

To investigate the role of C/EBPβ in muscle stem cells during cancer cachexia, we used a validated tissue culture model [53] in which subconfluent C2C12 myoblasts were incubated with conditioned medium (CM) from the human prostate cancers PC-3 and DU145, or with unconditioned media (UM) for 2 days prior to induction to differentiate (Fig 2A). Consistent with previous reports, incubation with CM from both cancers abrogated myogenesis, as evidenced by a reduction in the number and size of myosin heavy chain (MyHC) positive myotubes (Fig 2B) [53]. The fusion index (# nuclei in MyHC+ cells/# myocytes) was reduced ~60% by PC-3 medium and ~30% by DU145 medium as compared to controls (Fig 2C), The differentiation index (#nuclei in MyHC+ cells/total nuclei) was reduced approximately 40% in cells pre-treated with PC-3 medium, and ~30% for those treated with DU145 medium, as compared to controls (Fig 2C), suggesting that both the number and maturity of the myotubes was affected by a brief exposure to tumor-conditioned medium. In proliferating myoblasts, exposure to CM from both tumors stimulated C/EBPβ expression after two days, without affecting Pax7 expression (Fig 2D). The expression of MyoD was reduced in cells treated with DU145 CM, though not in cells treated with PC-3 medium (Fig 2D). After differentiation (day 7), pre-treatment with PC-3 conditioned medium inhibited myogenin and MyHC expression, consistent with impaired differentiation, without impacting MyoD expression (Fig 2E). Further, the mRNA expression of *Myod1*, *Myog* and neonatal *Myh* isoforms (*Myh1*, *Myh2*, *Myh8* and *Myh13*) were inhibited by exposure to PC-3 medium relative to cells exposed to unconditioned medium (Fig 2F). DU145 medium similarly reduced *Myog* and neonatal *Myh* isoform expression, without impacting *Myod1* expression.

### Inhibition of myogenesis by tumor-conditioned medium varies with ability to induce C/EBPβ in C2C12 and primary myoblasts

**Fig 3:**
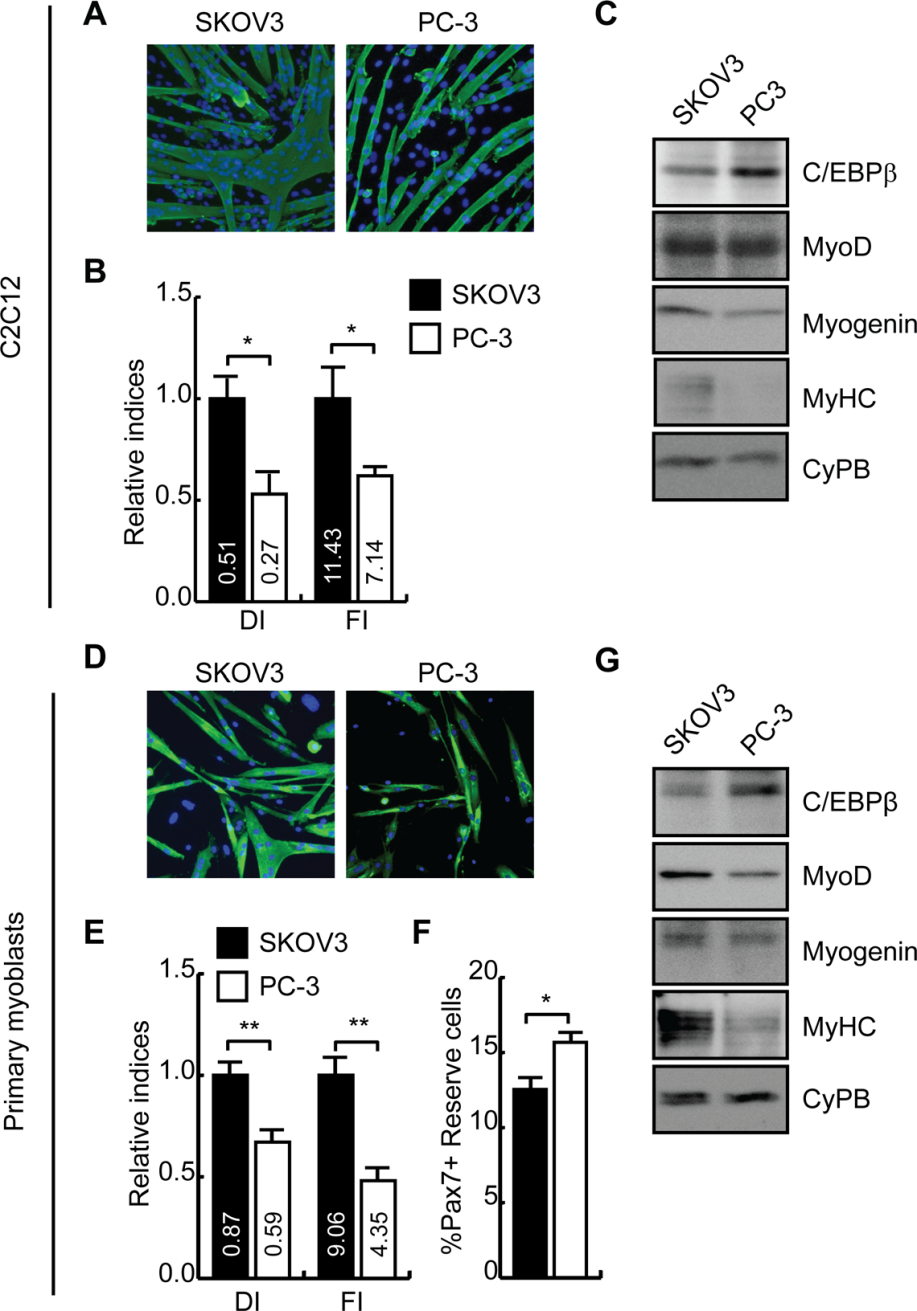
Inhibition of myogenesis correlates with induction of C/EBPβ expression in myoblasts. **(A)** Immunocytochemistry staining of myosin heavy chain expression in C2C12 myoblasts pre-treated with conditioned media from SKOV3 or PC-3 cells mixed 1:1 with fresh media, for 48hrs, and induced to differentiate for an additional 5 days. DAPI stains nuclei blue. **(B)** C2C12 cultures were induced to differentiate as in (A) and the differentiation and fusion indices were calculated as relative to SKOV3-treated cells. Actual values are shown in the respective bars. *p<0.05, n = 5. **(C)** C/EBPβ, and myogenic marker protein expression in C2C12 myoblasts treated as in (A). Cyclophilin B (CyPB) is shown as a loading control. **(D)** Immunocytochemistry staining of myosin heavy chain (MYH) expression in primary myoblasts pre-treated with conditioned media from SKOV3 or PC-3 cells mixed 1:1 with fresh media, for 48hrs, and induced to differentiate for an additional 48 hours in DMEM containing 10% horse serum. DAPI stains nuclei blue. **(E)** Primary myoblast cultures were induced to differentiate as in **(D)** and the differentiation and fusion indices were calculated as relative to SKOV3-treated cells. Actual values are shown in the respective bars. **p<0.01, n = 4. **(F)** Percentage of Pax7+ cells relative to total nuclei remaining in C2C12 cells cultured in conditioned medium and differentiated as in (D) as determined by immunocytochemistry. *p<0.05, n = 4. **(G)** C/EBPβ, and myogenic marker protein expression in primary myoblasts treated as in (D). Cyclophilin B (CyPB) is shown as a loading control.

Since conditioned medium would be expected to contain a mixture of growth factors and cytokines that can influence entry into myogenesis, we repeated the in culture cachexia model using conditioned medium from SKOV3 cells that only weakly stimulates C/EBPβ expression. While pre-treatment with SKOV3 medium allowed for robust differentiation, pre-treatment with PC-3 medium inhibited the formation of myotubes, decreasing the differentiation index by approximately 50% and the fusion index by ~40% (Fig 3A and 3B). Further, in accordance with the differences in C/EBPβ induction by SKOV3 and PC-3 CM, myogenin expression and MyHC expression was decreased in cells exposed to PC-3 medium as compared to SKOV3 controls (Fig 3C).

In agreement with the C2C12 model, pre-treatment of primary myoblasts with conditioned medium from the PC-3 tumor lead to the formation of fewer and smaller myotubes after 2 days in differentiation medium as compared to cells treated with conditioned medium from the SKOV3 tumor (Fig 3D). The differentiation index was decreased ~30% with PC-3 pre-treatment when compared to cells treated with SKOV3, while the fusion index was decreased ~50% in PC-3-treated myoblasts (Fig 3E). Further, the number of Pax7+ cells remaining in the cultures after differentiation, as determined using immunocytochemistry, was significantly increased in cultures pre-treated with PC-3 medium by approximately 25% as compared to SKOV3 controls (Fig 3F). As predicted, C/EBPβ levels were increased in PC-3 treated cells as compared to controls, and the expression of myogenin and myosin heavy chain were decreased. Interestingly, in contrast to our observations in the C2C12 model, primary myoblasts treated with PC-3 CM also demonstrated a decrease in MyoD protein expression, a finding consistent with our previous observations in this system (Fig 3G) [41, 42].

### C/EBPβ expression is increased in primary myoblasts isolated from cachectic mice

**Fig 4:**
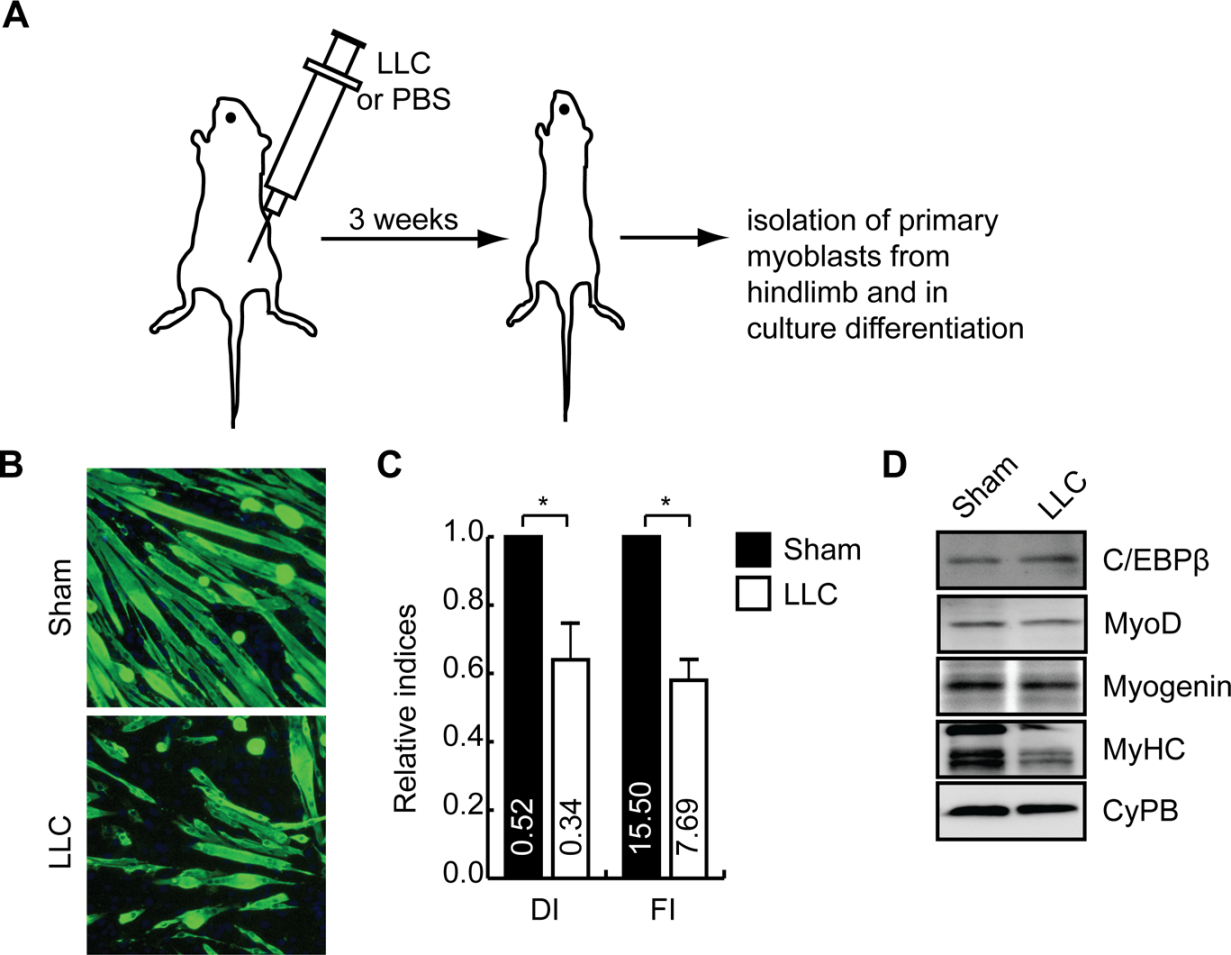
LLC tumor graft increases C/EBPβ expression in myoblasts and prevents myogenesis. **(A)** 5x10^5^ Lewis Lung Carcinoma (LLC) cells or PBS (Sham) was injected into the flank of C57BL/6 mice and allowed to engraft for 3 weeks to induce cachexia. **(B)** Immunocytochemistry for myosin heavy chain expression in primary myoblasts isolated from sham-injected and LLC-injected mice and differentiated for 2 days. **(C)** Differentiation (DI) and fusion (FI) indices from cultures isolated and differentiated as in (B). *p<0.05, n = 5. **(D)** Western analysis of C/EBPβ and myogenic marker expression in cells isolated from healthy or cachectic mice as in (A) and after culture expansion for 5 days, differentiated for 2 days. Cyclophilin B (CyPB) is a loading control.

To determine if the cancerous environment could stimulate C/EBPβ expression in myoblasts *in vivo*, we isolated muscle satellite cells from healthy and cachectic LLC tumor-bearing animals and differentiated them *ex vivo* (Fig 4A). Whereas SCs purified from healthy muscles differentiated efficiently, myoblasts isolated from cachectic animals produced fewer and smaller myotubes (Fig 4B). The differentiation index was reduced by 38% in cultures from cachectic animals and the fusion index was reduced 46% as compared to healthy controls (Fig 4C). As these cells were expanded in normal medium for 5 days before induction to differentiate, C/EBPβ levels were only mildly increased after differentiation, resulting in normalized MyoD expression (Fig 4D) and a small decrease in myogenin expression. Primary myoblasts removed from the cachectic mice also demonstrated a decrease in MyHC expression when compared to sham controls, consistent with the differentiation defect observed (Fig 4D). Hence, similar to our CM experiments, satellite cells originating from LLC tumor-grafted animals have increased C/EBPβ expression and a defect in differentiation that persists *ex vivo*.

### Cancer cachexia inhibits muscle regeneration in vivo

**Fig 5:**
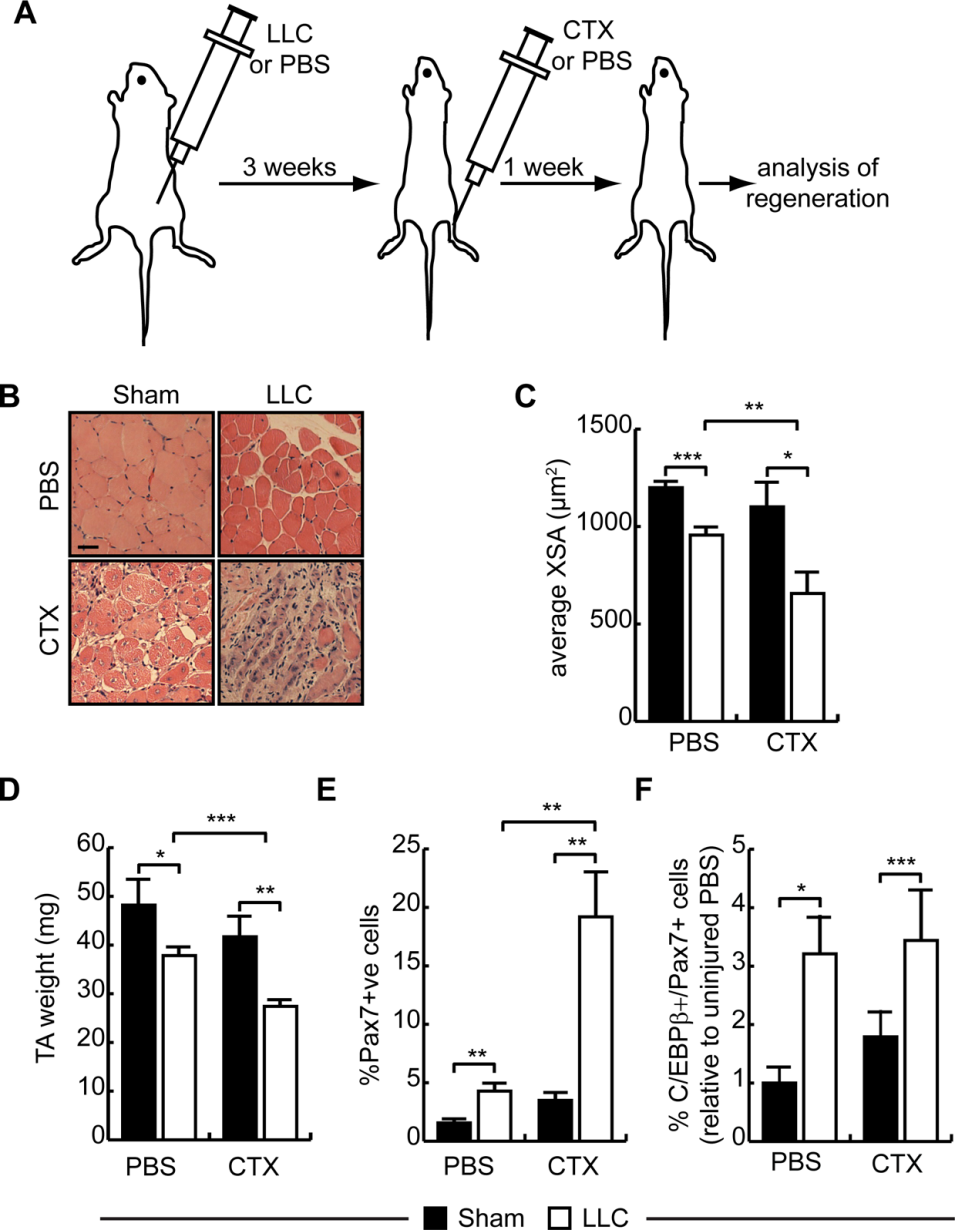
Defective muscle regeneration in cachectic animals after acute injury. **(A)** Schematic representation of the injury model. Cachexia was induced by engrafting LLC cancer cells subcutaneously and allowing them to grow for 3 weeks. Once cachexia was established, animals were injured by injecting cardiotoxin (CTX) into the TA muscle. Sham injury was achieved using PBS. Injury was allowed to repair for one week before analysis. **(B)** H&E-stained TA cross-sections from sham and LLC-injected mice as in (B) 7 days after CTX injury. The contralateral TA was injected with PBS alone. Scale bar = 20μm. **(C)** Average fiber XSA of PBS and CTX-injured sham and LLC mice as in (B). *p<0.05, **p<0.01, ***p<0.001, n = 4. **(D)** TA mass in PBS and CTX-injured sham and LLC mice as in (B). *p<0.05, **p<0.01, ***p<0.001, n = 5. **(E)** Percentage of Pax7+ cells (relative to total DAPI+ nuclei) in PBS and CTX-injured TA of sham and LLC animals. **p<0.01, ***p<0.001, n = 5. **(F)** Percentage of C/EBPβ+/Pax7+ cells (relative to uninjured sham animals) in PBS and CTX-injured TA of sham and LLC animals. *p<0.05, ***p<0.001, n = 3.

To assess skeletal muscle regenerative capacity in cachectic animals, we injured the left TA muscle with cardiotoxin (CTX) once cachexia was established and assessed repair one week later (Fig 5A). Seven days after injury, the TA muscle from healthy animals repaired efficiently with numerous myofibers with centrally located nuclei visible, whereas the injured TA of cachectic animals had extensive fibrosis and inflammatory cell infiltration with little evidence of regeneration (Fig 5B). Indeed, while repair restored fiber cross-sectional area to uninjured levels in healthy control mice, in cachectic mice, regenerating muscle fiber cross-sectional area was further reduced by 28% after injury, measuring only 59% of CTX-injured non-cachectic muscle (Fig 5C). Consistent with these observations, the TA weight of injured healthy animals at necropsy had returned to that of controls, whereas the weight of the injured TA in cachectic animals was only 73% of the uninjured cachectic control (Fig 5D).

We next scored the number of Pax7+ cells in TA cross-sections (Fig 5E). While approximately 1.6% of nuclei stained positively for Pax7 in the uninjured TA muscles of sham animals, this number reached 3.5% in uninjured LLC-bearing animals. Following CTX injury, Pax7+ cells increased in both sham and LLC animals, with a 2-fold increase for sham control animals as compared to the uninjured leg and a 4.5-fold increase over the uninjured leg in tumor-bearing animals to almost 20% of nuclei. Further, while injury did not affect the percentage of Pax7+/C/EBPβ+ satellite cells in healthy animals, in the tumor-bearing cohort, the percentage of double positive cells was increased in both uninjured and injured muscle (Fig 5F), consistent with the *ex vivo* analysis of satellite cells (Fig 4D). Taken together, these results suggest that cancer cachexia increases the Pax7+ compartment size and these cells have increased expression of C/EBPβ but reduced regenerative capabilities.

### Loss of C/EBPβ expression protects myoblasts from cachexia in culture

**Fig 6:**
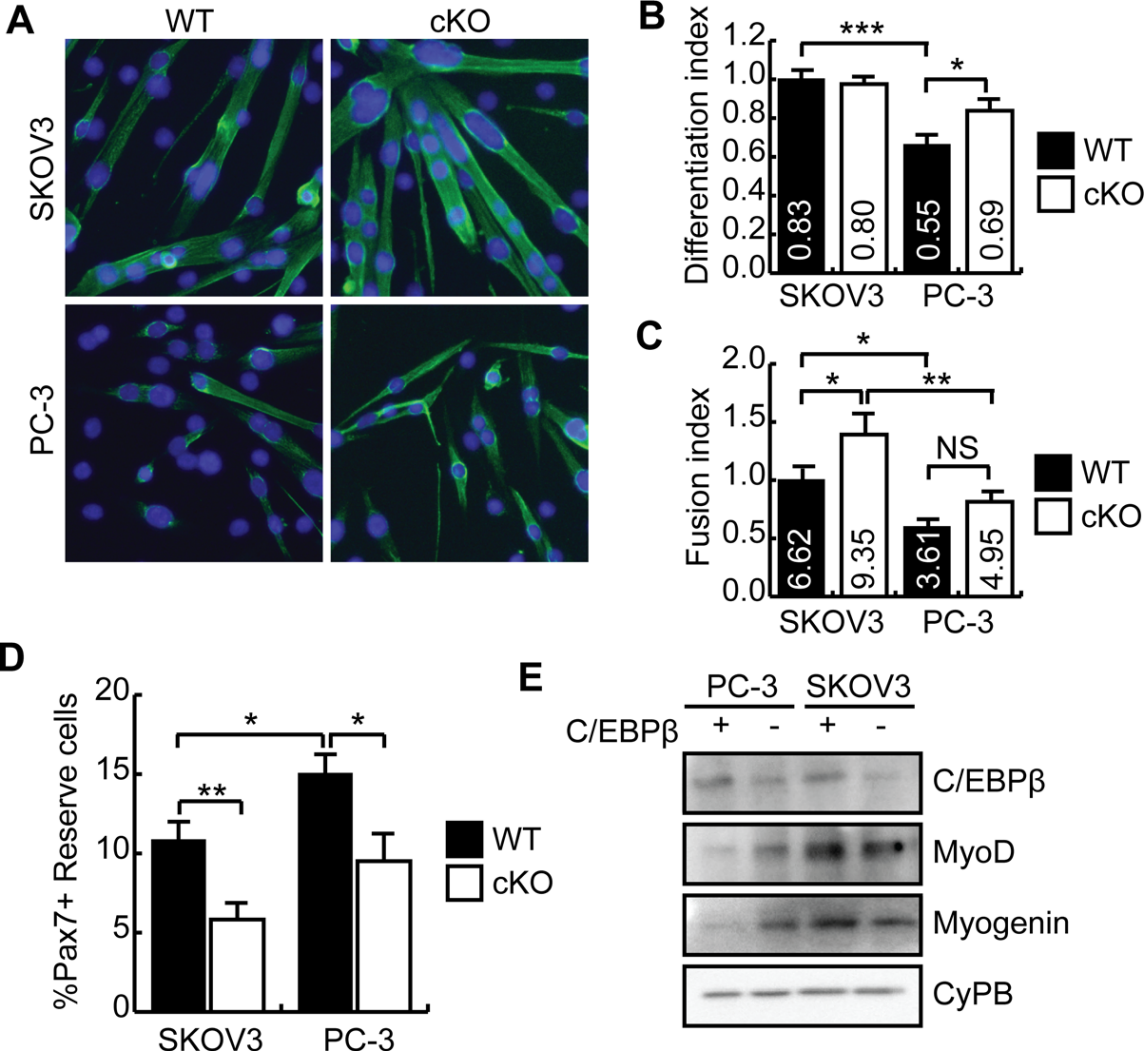
C/EBPβ null primary myoblasts are insensitive to conditioned medium from the PC-3 tumor. **(A)** Indirect immunofluorescence of myosin heavy chain (MyHC) expression in primary myoblasts isolated from *Cebpb* ^fl/fl^ *Pax7* ^+/+^ (WT) and *Cebpb* ^fl/fl^ *Pax7* ^CreER/+^ (cKO) mice and cultured with SKOV3 or PC-3 conditioned media for 2 days, and induced to differentiate for an additional 2 days. **(B)** Differentiation index (#myonuclei/#nuclei) of primary myoblasts cultures in (A). *p<0.05, ***p<0.001, n = 6. **(C)** Fusion index (#myonuclei/myotube) for cells differentiated as in (A). *p<0.05, **p<0.01, n = 6. **(D)** Percentage of Pax7+ cells relative to total nuclei in cultures from (A) following differentiation, designated as reserve cells. *p<0.05, **p<0.01, n = 6. **(E)** Western blot analysis of C/EBPβ and myogenic marker expression in primary myoblasts treated as in (A). Cyclophilin B (CyPB) is a loading control.

To confirm a role for C/EBPβ in the development of cancer cachexia, we isolated *Cebpb* ^fl/fl^ *Pax7* ^+/+^ (WT) and *Cebpb* ^fl/fl^ *Pax7* ^CreER/+^ (cKO) satellite cells, and pre-treated them with CM from the PC-3 tumor or the SKOV3 tumor (control) prior to induction to differentiate in low serum conditions. After 2 days in differentiation medium, the cultures were immunostained for MyHC expression (Fig 6A). While pre-treatment with PC-3 CM inhibited differentiation in WT myoblasts, loss of C/EBPβ expression rescued differentiation under these conditions (Fig 6A and 6B), suggesting that loss of C/EBPβ expression protects myoblasts from the cachectic milieu. Despite restoration of the differentiation index in cKO cultures, loss of C/EBPβ expression did not restore myotube size (fusion index) which was reduced following incubation with the PC-3 medium (Fig 6C). While treatment of WT cells with PC-3 medium increased the percentage of Pax7+ cells in the cultures, consistent with a reduction in the differentiation index, cultures lacking C/EBPβ had significantly fewer reserve cells after differentiation in both control and cachectic conditions (Fig 6D). While no differences in myogenic marker expression were observed when comparing WT and cKO cells in SKOV3 control medium, western analysis revealed that MyoD and myogenin expression were reduced inWT cells following pre-treatment with PC-3 medium (Fig 6E). However, in cKO cells, MyoD expression was increased following incubation with PC-3 medium as compared to WT and myogenin expression was restored to control levels (Fig 6E). Thus, loss of C/EBPβ in muscle satellite cells can rescue myogenesis in the presence of conditioned medium, without restoring myotube size.

## Discussion

C/EBPβ is a transcription factor implicated in numerous biological processes including cellular differentiation, cell cycle regulation, immune system function and cell survival [33, 54–59]. As such, C/EBPβ expression is influenced by many different pathways including cytokine signalling, growth factors, the proteasome and cAMP signalling [42, 60, 61]. Herein, we demonstrate that C/EBPβ expression can be stimulated by exposure to conditioned medium from human cancers, with varying efficiency. Although this conditioned medium contains likely cytokine mediators, the exact factors in the conditioned media driving C/EBPβ expression remain unknown and may be different for each cancer. Nonetheless, the stimulation of C/EBPβ expression in myoblasts correlated with the inhibition of myogenesis that could be reversed with loss of C/EBPβ.

Pre-treatment of proliferating myoblasts with conditioned medium was sufficient to inhibit myogenesis, even though differentiation was accomplished in medium free of pro-cachectic signals. These experiments thus separate the anti-myogenic effects of conditioned medium from the myotube atrophy effects that can also occur. Interestingly, the myogenic defect was recapitulated in primary myoblasts isolated from cachectic mice, even after expansion in growth medium not containing mouse serum or conditioned medium. It is conceivable that exposure to a cachectic milieu drives persistent epigenetic modification of loci involved in myogenesis that impair proper expression of proteins required for differentiation. Exposure to TNFα, a cytokine frequently associated with cachexia, has been shown to induce methylation of CpG islands within the MyoD locus, and the histone methylarginine transferases PRMT5 and WDR77/MEP50 can induce transcriptional repression of C/EBPβ target genes by inducing the symmetrical dimethylation of histone H4 arginine 3 [62, 63]. Given that primary myoblasts lacking C/EBPβ expression can differentiate normally following exposure to cachexia-inducing milieu, C/EBPβ is likely required for the patterning of these proposed epigenetic modifications. While differentiation following treatment with the PC-3 conditioned medium is restored with loss of C/EBPβ, the myotube size, measured as the fusion index, was not rescued. These results would suggest that pre-treatment with a cachectic medium prior to differentiation reduces the fusogenic capacity of the cells after differentiation, even in normal medium. As such, in addition to directly driving the atrophy of mature fibers and reducing their differentiation, cachexia can also impair fusion of successfully differentiated cells. While tumor resection can cure cachexia, it remains unknown whether regenerative responses recover fully or whether a defect persists once the cachectic environment resolves, and should be further explored.

The differentiation defect following treatment with PC-3 medium can be attributed to a failure to induce myogenin fully, which can be reversed by loss of C/EBPβ. In primary myoblasts, MyoD protein expression was also reduced by exposure to cachectic milieu, but was not observed in C2C12 cells. Indeed, C/EBPβ expression is lower in C2C12 when compared to primary myoblasts, and thus differences in C/EBPβ levels following exposure to conditioned medium may in itself explain the differential sensitivity of MyoD expression in the two systems [41]. C/EBPβ can inhibit MyoD’s ability to transactivate the myogenin promoter and the expression of myogenin was consistently downregulated in all models tested, suggesting that interference with MyoD function as a possible mechanism for the loss of myogenin expression in myoblasts pre-treated with conditioned medium [41].

MyoD protein expression is negatively regulated by C/EBPβ, without concomitant changes to *Myod1* mRNA levels [41]. Further, MyoD protein could not be rescued with inhibition of the proteasome [42]. Interestingly, loss of C/EBPβ expression does not increase MyoD expression under control conditions (SKOV3) when compared to WT cells though in these experiments, differentiation efficiency of the cultures is approximately 80%, making it more challenging to see increases in myogenic marker expression. However, in the absence of C/EBPβ after treatment with PC-3 medium, we do observe an increase in MyoD expression over that observed in WT cultures, suggesting that the loss of the C/EBPβ inhibitory mechanism is at play in this system.

While loss of C/EBPβ expression can improve differentiation after conditioned media pre-treatment, it remains unclear whether the inhibition of myogenesis in cancer cachexia contributes to the pathogenesis of the syndrome. Since cachexia also induces muscle injury [50], stimulation of the regenerative response would be predicted to be protective, and indeed, many reports have demonstrated that bolstering regeneration improves the muscle atrophy seen in cachexia [49, 50, 64]. Unfortunately, our results show that this response is inhibited by C/EBPβ expression, potentially exacerbating the muscle wasting. Nonetheless, mechanisms to stimulate myogenesis do improve muscle histology and lifespan in animal models suggesting that promotion of regeneration is an important therapeutic avenue for the treatment of cachexia.

## Materials and Methods

### Cell lines

C2C12 myoblasts and Lewis Lung carcinoma (ATCC) were cultured in DMEM supplemented with 10% fetal bovine serum (Gibco). PC-3 and DU145 prostatic adenocarcinoma cells (ATCC) were cultured in RPMI1640 supplemented with 10% fetal bovine serum and 2mM L-glutamine (Sigma). SKOV3 ovary adenocarcinoma and HCT116 colorectal carcinoma were cultured in McCoy media, MCF7 mammary gland adenocarcinoma were cultured in DMEM and A549 lung epithelia carcinoma were cultured in F-12K media, all supplemented with 10% fetal bovine serum.

Skeletal muscle primary myoblasts cells were isolated as described [65]. Lower hindlimb muscles of female C57BL/6 mice were dissected and digested with dispase and collagenase (Roche) at 37°C for 1–2 hrs until muscles were dissociated. After filtration and centrifugation, cells were pre-plated in DMEM containing 20% fetal bovine serum, 10% horse serum (Gibco) and penicillin/streptomycin (Wisent) at 37°C for 2hrs in a humidified incubator at 5% CO_2_. Supernatant was plated on matrigel-coated dishes (BD Biosciences) with media containing basic fibroblast growth factor [10ng/ml] and hepatocyte growth factor [2ng/ml] (Peprotech). Purity of resulting cultures was monitored using Pax7 immunofluorescence and deemed to be consistently over 90% pure.

### Cellular differentiation and conditioned media

C2C12 myoblasts were differentiated in DMEM containing 2% horse serum at 80% confluency for 5 days. Primary myogenic stem cells were differentiated in DMEM with 2% fetal bovine serum and 10% horse serum at 80% confluency for 2 days. For the in culture analysis of cancer cachexia, confluent human prostate cancer cell lines were incubated with fresh growth medium for 48hrs, and the conditioned medium was filtered through a 0.45μm syringe filter and mixed 1:1 with fresh media before addition to proliferating C2C12 or primary myoblasts. The fusion index was calculated by scoring the average number of myonuclei per myotube. Differentiation index was calculated by scoring the average number of myonuclei divided by the total number of nuclei.

### Western Analysis and Antibodies

Whole cell lysate were prepared in IPH buffer (50mM Tris pH 7.5, 150mM NaCl, 0.5% NP-40, 5mM EDTA, 1mM DTT and 1X protease inhibitor cocktail) and briefly sonicated on ice. To assess expression of myoblast and myotube markers, the following antibodies were used: mouse anti-MyoD (SC-32758, 1/500), mouse anti-myogenin (SC-12732, 1/500), rabbit anti-C/EBPβ (SC-150, 1/500) all from Santa Cruz Biotechnology, mouse anti-Pax7 (pax7, 1/200) and mouse myosin heavy chain (MF20, 1/100) both from DSHB. Rabbit anti-Cyclophilin B (Abcam, 1/10,000) and mouse anti-β-actin (Sigma, 1/10,000) were used as loading controls. Secondary HRP-conjugated antibodies used were donkey anti-rabbit (NA934, 1/2,000) and sheep anti-mouse (NA931, 1/2,000) both from Amersham GE Healthcare Life Sciences. Chemiluminescence images were captured using the Luminescent Image Analyzer LAS-4000 (Fujifilm Life Science).

### Indirect Immunofluorescence

For immunocytochemistry, cells were washed, fixed in 2% paraformaldehyde, permeabilized with 0.5% Triton X-100 and blocked 1 hour in 5% normal donkey serum. Following washes, mouse anti-MyHC (MF20, 1/200), mouse anti-Pax7 (Pax7, 1/100) both from DSHB, or rabbit anti-MyHC (SC-20641, 1/100) primary antibody was added and incubated at 4°C. Secondary antibodies used were: Alexa488-conjugated donkey anti-rabbit (711-546-152, 1/500), biotin-conjugated donkey anti-mouse (715-066-150, 1/500), Cy3-conjugated streptavidin (016-160-084, 1/1,000) all from Jackson Immuno-Research. Nuclei were identified by DAPI staining.

Indirect immunofluorescence was performed on frozen sections of TA muscle from C57BL/6 mice. 8μm-thick sections were dehydrated 30min at 37°C, fixed in 4% paraformaldehyde and antigen-retrieval was perform by incubating sections at 92°C in citrate buffer (10mM citric acid, 0.05% Tween-20, pH 6.0) for 20min. Primary antibodies used were rabbit anti-C/EBPβ (SC-150, 1/200) from Santa Cruz Biotechnology, anti-Pax7 (pax7, 1/100) from DSHB. Secondary antibody labelling was done as above. For image acquisition, pictures were taken on a DMI3000B epifluorescence microscope (Leica) using infinity 3 (Lumenera) camera. For processing, individual pictures were level adjusted uniformly and pasted on a single color channel in Adobe Photoshop.

### Quantitative RT-PCR

Total cell RNA was purified with the RNAeasy kit (Qiagen). 1μg of purified RNA was DNase digested for 30 minutes at 37°C (Ambion) and cDNA was synthesized using the iScript kit (Bio-Rad). cDNA was PCR amplified on a Stratagene MX3005p real-time thermocycler using a iTaq universal SYBR Green kit (Bio-Rad). Relative transcript expression was computed using the ΔΔCt method relative to the expression of 18S rRNA. The following oligonucleotides primers were used: *Cebpb*-f: 5’-TCGAACCCGCGGACTGCAAG-3’, *Cebpb*-r: 5’-CGACGACGACGTGGACAGGC-3’, *Myod1*-f: 5’-TGGCATGATGGATTACAGCG-3’, *Myod1*-r: 5’-CCACTATGCTGGACAGGCAGT-3’, *Myog*-f: 5’- ATCGCGCTCCTCCTGGTTGA-3’, *Myog*-r: 5’-CTGGGGACCCCTGAGCATTG-3’, *Myh*-f: 5’-TCGCTGGCTTTGAGATCTTT-3’, *Myh*-r: 5’-ACGAACATGTGGTGGTTGAA-3’ *18S*-f: 5’-CGCCGCTAGAGGTGAAATC-3’, *18S*-r: 5’-CCAGTCGGCATCGTTTATGG-3’ for *mus musculus* samples, and for *homo sapiens* samples: *IL1B*-f: 5’- TGCTCTGGGATTCTCTTCAGC-3’, *IL1B*-r: 5’-AAGTCATCCTCATTGCCACTGT-3’, *18S*-f: 5’- GTCTAAGTACGCACGGCCGGT-3’, *18S*-r: 5’-CAGCGCCCGTCGGCATGTATT-3’. All primers were designed using the Primer3 algorithm.

### LLC-tumor graft, cardiotoxin muscle injury and knockout animals

Six week-old C57BL/6 female mice (Charles River) were injected subcutaneously with 5x10^5^ LLC cells or with PBS. Tumors were allowed to grow for a total of 4 weeks after grafting with wellness monitoring every two days at which time tumor size was assessed. At experimental endpoint, the tumor mass was on average 1.34 +/- 0.21g. While only female mice were used for analysis, muscle wasting was comparable in male mice (S1 Fig). For TA-muscle injury, 30μl of 10μM cardiotoxin (Latoxan) was injected intramuscularly in the tibialis anterior of mice anesthetized with isofluorane three weeks after tumor grafting and allowed to repair for an additional week. The *Cebpb* ^fl/fl^ *Pax7* ^+/+^ (WT) and conditional null (cKO, *Cebpb* ^fl/fl^ *Pax7* ^CreER/+^) mice have been described previously [41]. Mice were housed in regular animal facility at the University of Ottawa and with free access to water and food. All animal work was performed in accordance with the guidelines set out by the Canadian Council on Animal Care and was approved by the University of Ottawa Animal Care Committee.

### Statistical significance

All data represents the average of indicated number of independent experiment performed. Error bars represent the standard error of the mean. Two-tailed t-test or two-way ANOVA with post-hoc test were used to calculate p-values.

## Supporting Information

S1 Fig Male mice develop cachexia after engraftment with the LLC tumor. **(A)** Cachexia was induced by engrafting LLC cancer cells subcutaneously into male mice and allowed to grow for 4 weeks. Average fiber XSA was calculated from H&E-stained TA cross-sections from sham and LLC-injected mice, *p<0.05, n>4. **(B)** TA mass in sham and LLC mice as in (A). *p<0.05, n>4. **(E)** Percentage of Pax7+ cells (relative to total DAPI+ nuclei) in TA muscle of sham and LLC animals. *p<0.05, n>4.
(TIF)
