# Supplementary figures and images for: Expression of CCAAT/Enhancer Binding Protein Beta in Muscle Satellite Cells Inhibits Myogenesis in Cancer Cachexia
Source: PLoS One. 2015 Dec 28;10(12):e0145583. doi: 10.1371/journal.pone.0145583 (PMC4692409; doi:10.1371/journal.pone.0145583)

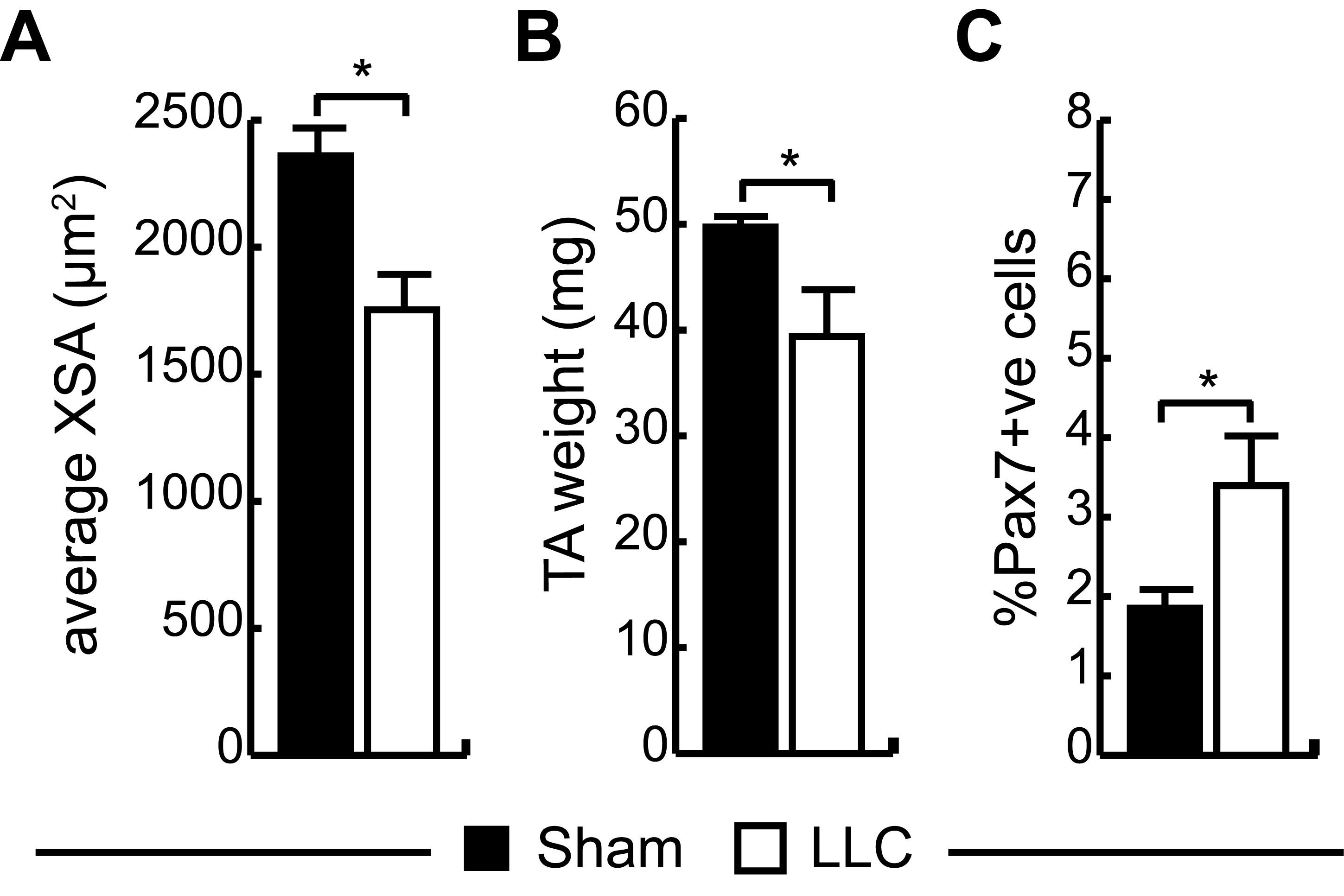

Supplement: S1 Fig — (A) Cachexia was induced by engrafting LLC cancer cells subcutaneously into male mice and allowed to grow for 4 weeks. Average fiber XSA was calculated from H&E-stained TA cross-sections from sham and LLC-injected mice, *p<0.05, n>4. (B) TA mass in sham and LLC mice as in (A). *p<0.05, n>4. (E) Percentage of Pax7+ cells (relative to total DAPI+ nuclei) in TA muscle of sham and LLC animals. *p<0.05, n>4. (TIF) [file pone.0145583.s001.tif]
